# Examining Social Vulnerability and the Association With COVID-19 Incidence in Harris County, Texas

**DOI:** 10.3389/fpubh.2021.798085

**Published:** 2022-01-05

**Authors:** Guillermo A. Tortolero, Marcia de Oliveira Otto, Ryan Ramphul, Jose-Miguel Yamal, Alison Rector, Michael Brown, Melissa F. Peskin, Dania Mofleh, Eric Boerwinkle

**Affiliations:** ^1^Department of Epidemiology, Human Genetics and Environmental Sciences, School of Public Health, The University of Texas Health Science Center at Houston, Houston, TX, United States; ^2^Department of Biostatistics and Data Science, School of Public Health, The University of Texas Health Science Center at Houston, Houston, TX, United States; ^3^Department of Health Promotion and Behavioral Sciences, School of Public Health, The University of Texas Health Science Center at Houston, Houston, TX, United States

**Keywords:** epidemiology, vulnerability, geography, COVID-19, infectious disease

## Abstract

Studies have investigated the association between social vulnerability and SARS-CoV-2 incidence. However, few studies have examined small geographic units such as census tracts, examined geographic regions with large numbers of Hispanic and Black populations, controlled for testing rates, and incorporated stay-at-home measures into their analyses. Understanding the relationship between social vulnerability and SARS-CoV-2 incidence is critical to understanding the interplay between social determinants and implementing risk mitigation guidelines to curtail the spread of infectious diseases. The objective of this study was to examine the relationship between CDC's Social Vulnerability Index (SVI) and SARS-CoV-2 incidence while controlling for testing rates and the proportion of those who stayed completely at home among 783 Harris County, Texas census tracts. SARS-CoV-2 incidence data were collected between May 15 and October 1, 2020. The SVI and its themes were the primary exposures. Median percent time at home was used as a covariate to measure the effect of staying at home on the association between social vulnerability and SARS-CoV-2 incidence. Data were analyzed using Kruskal Wallis and negative binomial regressions (NBR) controlling for testing rates and staying at home. Results showed that a unit increase in the SVI score and the SVI themes were associated with significant increases in SARS-CoV-2 incidence. The incidence risk ratio (IRR) was 1.090 (95% CI, 1.082, 1.098) for the overall SVI; 1.107 (95% CI, 1.098, 1.115) for minority status/language; 1.090 (95% CI, 1.083, 1.098) for socioeconomic; 1.060 (95% CI, 1.050, 1.071) for household composition/disability, and 1.057 (95% CI, 1.047, 1.066) for housing type/transportation. When controlling for stay-at-home, the association between SVI themes and SARS-CoV-2 incidence remained significant. In the NBR model that included all four SVI themes, only the socioeconomic and minority status/language themes remained significantly associated with SARS-CoV-2 incidence. Community-level infections were not explained by a communities' inability to stay at home. These findings suggest that community-level social vulnerability, such as socioeconomic status, language barriers, use of public transportation, and housing density may play a role in the risk of SARS-CoV-2 infection regardless of the ability of some communities to stay at home because of the need to work or other reasons.

## Introduction

The United States (US) has been severely affected by SARS-CoV-2, but cases in the US are not evenly distributed across the population (1). Emerging studies suggest that social vulnerability and inability to stay at home play a major role in increased cases of SARS-CoV-2 within a community. However, few studies have assessed these factors simultaneously (2–9). Social vulnerability is the degree to which a community exhibits social conditions that may affect their ability to prevent serious injury, illness, or loss in the event of a disaster (10). CDC created the Social Vulnerability Index (SVI) for local public health organizations to assess and prioritize census tracts that may be particularly vulnerable to disasters such as a pandemic (10). Though CDC's SVI was not constructed with a pandemic in mind, the SVI can be very useful in identifying vulnerable populations, poverty, and living conditions which may make populations more susceptible to communicable diseases such as SARS-CoV-2 (10). Sheltering in place has been a primary prevention strategy to mitigate the spread of SARS-CoV-2. Research shows that staying at home is one of the most effective strategies in controlling the pandemic (11, 12). However, disadvantaged communities may be less likely to be able to stay at home because of financial constraints; the inability to work from home compared to higher wage-earners; being a member of the essential workforce; having fewer savings, and having to work or risk losing income (11, 12).

A growing number of studies have investigated the association between social vulnerability and SARS-CoV-2 incidence (13–16). However, these studies have four important limitations: (1) few studies examine smaller geographic units such as census tracts; (2) few studies examine geographic regions with large numbers of Hispanic/Latino, Black, and Asian populations; (3) few studies control for testing rates; and, (4) even fewer incorporate stay-at-home measures into the analysis. Controlling for stay-at-home measures can provide a more precise picture of how social vulnerability and staying at home impact SARS-CoV-2 incidence (4, 9). Our study objective is to overcome these limitations and extend this work by examining the relationship between social vulnerability, proportion of the population staying at home, and SARS-CoV-2 infection rates in Harris County, Texas, by census tract.

## Methods

### Setting

This study analyzed SARS-CoV-2 incidence data reported in 783 census tracts in Harris County, Texas. Harris County has over 4.7 million people and is one of the most diverse regions in the country, with 44% of the population identifying as Hispanic or Latino, 29% identifying as White, 20% identifying as Black, and 7% identifying as Asian (17). The County has a median annual income of $61,705 and a poverty level of 15%, higher than the national average of 13% (17).

### Outcome: SARS-CoV-2 Incidence

For this ecological study, census tract-level SARS-CoV-2 incidence reported to Harris County Public Health Department and the City of Houston Public Health Department from May 15 to October 1, 2020, was analyzed. The date of diagnosis of a SARS-CoV-2 case was defined as the first positive test result. The dependent variable, SARS-CoV-2 incidence, was calculated as both a case count and a rate per 1,00,000 using the census tract population estimated by the US Census 2019 American Community Survey 5-year estimates. SARS-CoV-2 incidence was calculated over the entire time period.

### Exposure: Social Vulnerability Index

Independent variables consisted of an overall social vulnerability index and four social vulnerability themes. The social vulnerability index used in this study was acquired through publicly-available data from the CDC's 2018 Social Vulnerability Index (SVI) (10). The CDC uses US Census data to determine the social vulnerability of every census tract. The SVI ranks each census tract on 15 social factors, including poverty, lack of vehicle access, and crowded housing, and groups them into four indices (themes): socioeconomic status, household composition/disability, minority status/language, and housing type/transportation. CDC offers the SVI for the United States and for each individual state. For state data, census tracts are ranked against other tracts within the state. Texas rankings were used for these analyses (10). CDC calculates the SVI by summing scores and converting these scores into a percentile rank ranging from 0 to 1, with higher scores denoting greater vulnerability (10). SVI measures were initially described by quintiles and then analyzed as a continuous variable in the negative binomial regression models. The SVI index was rescaled to an integer value by multiplying the SVI percentage by 10 so that the index ranged from 0 to 10 for ease of interpretation. A 1-unit change in the results refers to a decile (0.1) increase on the original scale.

### Covariates

The percent of individuals fully staying-at-home was acquired from SafeGraph, a company that aggregates anonymized smartphone location data in the United States. SafeGraph compiles individuals' cell phone data into aggregate measures on the census tract-level over time and then calculates the proportion of smartphone users who spent all day at home for each date based on inferring the user's overnight location during the previous six weeks (18). Proportion staying-at-home was calculated by cumulatively averaging the measures over the study time by census tract. The percent time at home measure was initially described and mapped by quintiles and analyzed as continuous variables in the models (19). These data correlates well with other smartphone location data, with the Gallup survey data on staying-at-home measures, and workplace visits reported by Google COVID-19 Community Mobility Reports (20).

Population estimates were acquired from the US Census 2019 American Community Survey 5-year estimates. The testing data was acquired from the Harris County Public Health Department and the City of Houston Public Health Department. All verified tests were used regardless of type (molecular or antigen). Testing rates were calculated per census tract by dividing the number of tests from May 15 to October 1, 2020 by the population within a census tract. The distribution of the testing rate was examined and subsequently transformed by taking the log.

### Analysis

This analysis included 783 census tracts in Harris County. Descriptive statistics of all variables were calculated. A bivariate map of SARS-CoV-2 incidence and the overall SVI by census tract was created to visualize the association between these two variables. A Kruskal-Wallis test was conducted to examine differences in incidence rates among the overall SVI quintiles, SVI theme quintiles, and the percent time at home measure quintiles. Correlations were performed for the association between all variables. Testing rates by census tract were examined and the association between testing rates, SVI, and SARS-CoV-2 incidence were analyzed. A negative binomial regression (NBR) model was used to analyze variations in SARS-CoV-2 incidence counts across census tracts. This model was used to handle the overdispersion of count-based data containing only non-negative integer values and consider the independent explanatory neighborhood-level characteristics (15, 21). Since case count data was used instead of incident rates, all models controlled for the population size. The log of population size and testing rate were used. Multivariable negative binomial regressions were used to examine the relationship between each individual SVI theme and SARS-CoV-2 incidence counts. The analysis plan was approached in steps. First, NBR models were conducted for each SVI theme controlling for the log of population and log of testing rates. Next, NBR models were performed for each SVI theme independently while controlling for the log of population, log of testing rates, and median percent time fully at home. The final NBR model included all four SVI themes controlling for the log of population, log of testing rates, and median percent time fully at home. The incidence risk ratio was calculated by exponentiating the regression coefficient and is interpreted as an increase or decrease in the risk of SARS-CoV-2 incidence associated with a one unit change in the independent variable (21). All models examined SARS-CoV-2 incidence between May 15 and October 1, 2020; this time period represents the second wave of the pandemic in Harris County. Significance testing is at *p* < 0.05. Data were analyzed using STATA 15.0 statistical software (StataCorp LLC) and ArcGISPro 2.8 (Environmental Systems Research Institute, Inc., California, USA). This study was reviewed by The University of Texas Health Science Center at Houston Committee for the Protection of Human Subjects.

## Results

**Figure 1:**
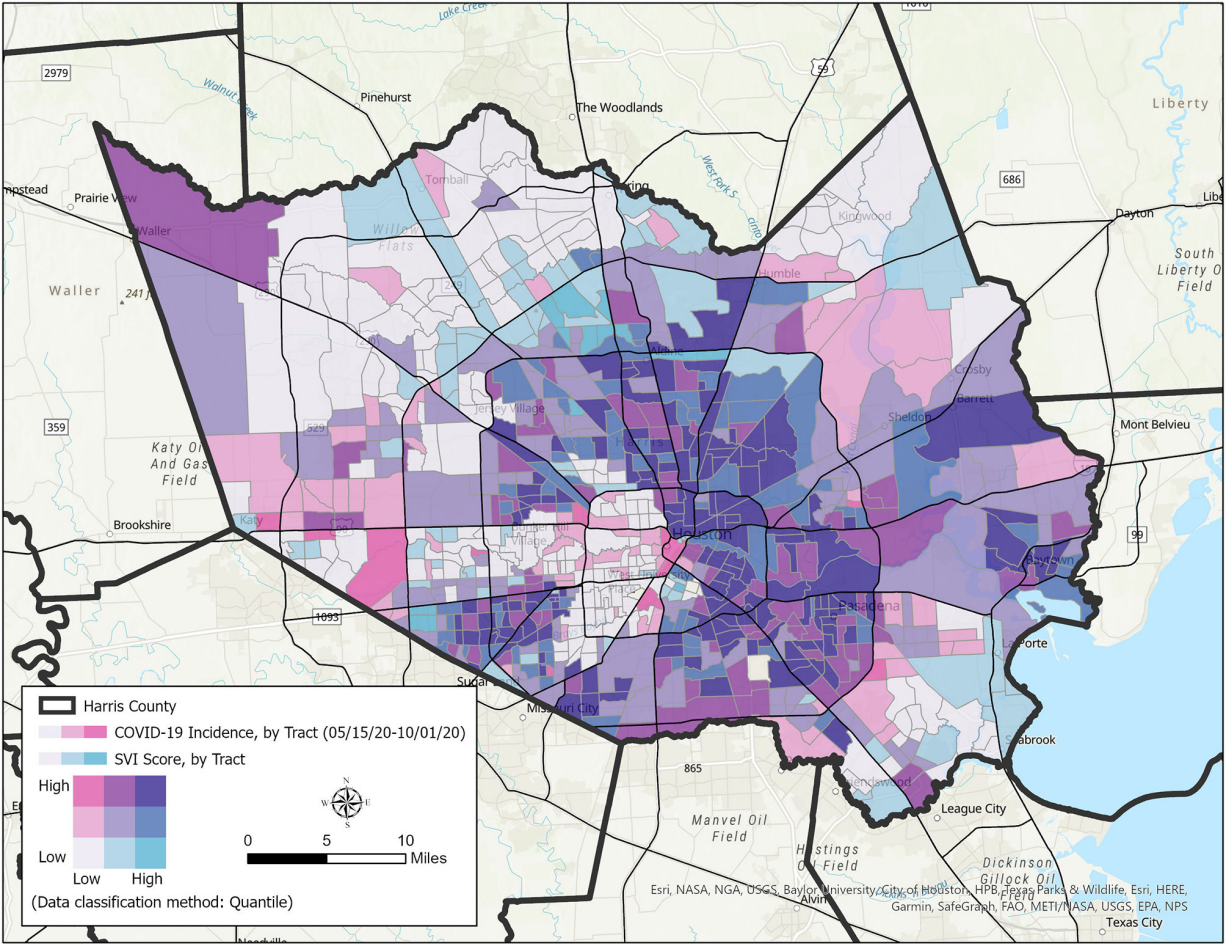
Bivariate map of SARS-CoV-2 incidence and social vulnerability index.

Between May 15 and October 1, 2020, there were 140,853 incident cases of SARS-CoV-2 within the Harris County Public Health Department and City of Houston Health Department jurisdictions. Testing rates by census tract ranged from 1,535.433 to 26,105.210 per 100,000 population in the studied census tracts (Table 1). The incidence of SARS-CoV-2 ranged from 433 to 9,791 per 100,000 population in the studied census tracts. The overall SVI, the socioeconomic status theme, the minority status/language theme, and the housing type/transportation theme were significantly associated with the percent-at-home measure. However, the SVI measures explained only a small amount of variance in the percent staying-at-home measure. Figure 1 displays a bivariate map of SARS-CoV-2 incidence and the overall SVI by census tract. A non-random pattern of increased SARS-CoV-2 incidence was observed in Harris County census tracts. Table 2 displays the distribution of SARS-CoV-2 incidence rates among SVI quintiles, and the median percent time completely at home quintiles. There were significant differences in the incidence rates of SARS-CoV-2 by quintiles for the overall SVI and its themes, with higher incidence rates for more vulnerable census tracts.

**Table 1:** Descriptive statistics of study variables for Harris County, Texas, May 15 to October, 2020 (*n* = 783).

	**Mean**	**Standard deviation**	**Median**	**Range**
Incidence per 1,00,000	2,873.167	1,230.145	2,812.384	433.189–9,791.332
Testing per 1,00,000	7,920.740	4,416.743	7,427.809	1,535.433–26,105.210
SVI	5.266	3.087	5.636	0.015–9.942
Socioeconomic	5.328	3.108	5.794	0.002–9.964
Household composition/ Disability	4.097	2.606	3.800	0.044–9.975
Minority status/Language	6.410	2.749	7.093	0.237–9.990
Housing type/Transportation	5.000	2.923	4.999	0.056–10.000
Median percent time completely at home	32.310	4.134	32.228	15.196–47.861

*SVI, Social Vulnerability Index*.

**Table 2:** Descriptive characteristics of SVI themes and stay-at-home measure by incidence rate per 1,00,000, Harris County, Texas, May 15 to October, 2020.

**Measures by quintiles**	**Median cumulative incidence rate per 1,00,000**	**Range of cumulative incidence rate per 1,00,000**	***P*-value**
SVI			<0.001
Q1	1,696.93	433.19, 4,116.95	
Q2	2,218.62	754.83, 5,905.84	
Q3	3,122.98	1,348.75, 7,391.49	
Q4	3,587.38	1,650.86, 9,791.33	
Q5 (Most vulnerable)	3,747.09	1,483.68, 7,099.70	
Socioeconomic theme			<0.001
Q1	1,717.76	433.19, 5,137.07	
Q2	2,310.39	622.41, 9,791.33	
Q3	2,946.15	754.83, 6,524.11	
Q4	3,642.40	1,483.68, 7,391.49	
Q5 (Most vulnerable)	3,755.22	1,580.44, 7,099.70	
Household Composition/Disability theme			<0.001
Q1	2,247.40	636.09, 7,391.49	
Q2	2,481.50	433.19, 6,524.11	
Q3	2,936.40	457.95, 5,703.97	
Q4	3,170.17	773.69, 6,123.70	
Q5 (Most vulnerable)	3,536.49	1,173.48, 9,791.33	
Minority status/Language theme			<0.001
Q1	1,682.32	433.19, 5,905.84	
Q2	2,329.11	1,027.40, 9,791.33	
Q3	2,922.41	1,039.36, 5,613.43	
Q4	3,597.05	1,483.68, 7,391.49	
Q5 (Most vulnerable)	3,841.43	1,587.82, 7,063.81	
Housing type/transportation theme			<0.001
Q1	1,965.93	433.19, 5,042.02	
Q2	2,562.11	478.32, 6,899.72	
Q3	2,988.70	852.48, 7,391.49	
Q4	3,194.03	1,072.21, 5,418.38	
Q5 (Most vulnerable)	3,660.81	1,352.34, 9,791.33	
Median percent time completely home			0.008
Q1	2,650.68	433.19, 9,791.33	
Q2	3,117.34	754.83, 7,099.70	
Q3	2,932.07	537.46, 7,063.81	
Q4	2,895.26	638.90, 5,950.03	
Q5 (Most Protected)	2,770.11	693.76, 5,418.38	

*SVI, Social Vulnerability Index; Q, quintile*.

The initial NBR models of the social vulnerability measures indicated that all SVI themes were significantly associated with cumulative SARS-CoV-2 incidence after controlling for population and testing rates (Table 3). For every unit increase in the overall SVI index, the incidence of SARS-CoV-2 increased 1.090 times (95% CI, 1.082, 1.098). The socioeconomic status theme was also associated with a 1.090 times (95% CI, 1.083, 1.098) increase in the incidence of SARS-CoV-2 for every unit increase. Similarly, the minority status/language theme was associated with a 1.107 (95% CI, 1.098, 1.115) increase in the incidence of SARS-CoV-2 for every unit increase. The household composition/disability theme, and the housing type/transportation theme were associated with ~1.060 times and 1.057 times increase, respectively, in the incidence of SARS-CoV-2 for every unit increase. After adding the staying-at-home measure to each model, the association between the SVI themes and SARS-CoV-2 incidence did not significantly change.

**Table 3:** The association between social vulnerability and incidence of SARS-CoV-2 after controlling for covariables, Harris County, Texas, May 15 to October, 2020 (*n* = 783).

	**Models of each SVI theme controlling for testing rate**				**Models of each SVI theme controlling for testing rate and stay-at-home measure**			
	**IRR**	**95% CI**		***p*-value**	**IRR**	**95% CI**		***p*-value**
SVI	1.090	1.082	1.098	<0.001	1.093	1.085	1.101	<0.001
Socioeconomic	1.090	1.083	1.098	<0.001	1.094	1.087	1.101	<0.001
Household composition/Disability	1.060	1.050	1.071	<0.001	1.060	1.050	1.071	<0.001
Minority status/Language	1.107	1.098	1.115	<0.001	1.114	1.106	1.122	<0.001
Housing type/Transportation	1.057	1.047	1.066	<0.001	1.058	1.049	1.068	<0.001

*SVI, Social Vulnerability Index; IRR, incident risk ratio; CI, confidence interval*.

In the multivariable model (Table 4) after controlling for testing rate, the staying-at-home measure, and entering all SVI themes, the socioeconomic theme and the minority status/language theme remained significantly associated with SARS-CoV-2 incidence. The minority status/language theme was strongly associated with SARS-CoV-2 incidence with a 1.078 (95% CI, 1.063, 1.093) increase in the incidence of SARS-CoV-2 for every unit increase. The household composition/disability theme and the housing type/transportation theme were no longer significantly associated with SARS-CoV-2 incidence after taking into account the other SVI themes, testing rates, and the staying-at-home measure.

**Table 4:** The multivariable association of all social vulnerability themes and incidence of SARS-CoV-2 after controlling for stay-at-home measure and testing rate, Harris County, Texas, May 15 to October, 2020 (*n* = 783).

	**IRR**	**95% CI**		***p*-value**
Socioeconomic	1.031	1.015	1.046	<0.001
Household composition/	1.078	0.997	1.016	0.175
Disability				
Minority status/Language	1.078	1.063	1.093	<0.001
Housing type/ Transportation	1.001	0.993	1.010	0.749

*IRR, incident risk ratio; CI, confidence interval*.

## Discussion

In this ecological study of Harris County census tracts between May 15 and October 1, 2020, we found that CDC's social vulnerability index and all four SVI themes were positively associated with SARS-CoV-2 incidence, even after controlling for testing rates and the stay-at-home measure. The magnitude of the associations of all four themes was substantial and remained positively associated with SARS-CoV-2 incidence, even after controlling for testing rates and the stay-at-home measure. After controlling for testing rates, the staying-at-home measure, and all SVI domains simultaneously, the socioeconomic theme and the minority status/language theme remained strongly associated with SARS-CoV-2 incidence, while the household composition/disability theme and the housing type/transportation theme did not.

This study adds to the growing body of literature seeking to understand community-level social determinants associated with SARS CoV-2 incidence. Other studies using CDC's SVI at both the census tract and county-levels had similar findings. Studies of US counties have found significant associations between SVI and SARS-CoV-2 incidence rates (14, 16). In a study that analyzed census tracts in Louisiana, Biggs and colleagues found a 52% increase (IRR, 1.52; 95% CI, 1.40–1.65) in SARS CoV-2 incidence per 0.01 increase in the overall SVI and a significant association for each of the SVI domains (13).

Socioeconomic inequalities have historically been strongly associated with disease incidence during pandemics, such as the Spanish influenza pandemic of 1918–1919 (22). The association between the socioeconomic status and SARS CoV-2 incidence in the present study is consistent with previous studies (13, 14, 16). For example, Karmakar and colleagues found an 11% increase in SARS CoV-2 incidence for every 0.1 increase in the socioeconomic status theme in all United States counties. Khazanchi et al. reported that the most vulnerable counties for the socioeconomic status theme had a 42% increase in SARS CoV-2 incidence compared to those in the least vulnerable counties (16). Biggs et al. found a 32% increase in SARS CoV-2 incidence for every 0.01 increase in the socioeconomic status theme among Louisiana census tracts (13). These findings are consistent with the present study.

Our study found significant associations between housing type/transportation and SARS CoV-2 incidence. Studies using data on both the county and census tract levels have found significant yet small associations between housing type/transportation and SARS CoV-2 incidence (13, 14, 16). These findings may suggest that housing type and transportation are important factors that increase one's risk of SARS CoV-2 exposure, but that other SVI factors are more influential to one's actual risk.

The SVI theme that was most strongly associated with SARS CoV-2 incidence in this study was minority status/language. This study's findings on the strong association between the minority status/language theme and SARS CoV-2 incidence are consistent with other studies. A study of US counties found that for every 0.1 increase in the minority status/language domain was associated with a 21.7% increase in SARS-CoV-2 incidence rate (IRR, 1.22; 95% CI, 1.20–1.23; *P* < 0.001) (14). Similarly, another study found that those in the most vulnerable counties for the minority status/language domain had a 4.94-fold greater risk (IRR, 4.94; 95% CI, 3.91–6.24) of SARS CoV-2 incidence (16). The strong association between the minority status/language domain and SARS CoV-2 incidence could possibly be explained through multiple routes but needs further exploration (23). Racial/ethnic disparities are often explained by socioeconomic, educational, systemic racism, structural inequalities, and housing differences. Some researchers have suggested that racial/ethnic disparities in SARS CoV-2 incidence may be related to the racial distribution of work in essential industries and the inability for these workers to social distance or stay at home (24). Though findings suggest that racial/ethnic minority populations have a greater risk for SARS CoV-2 infection, this is not due to some inherent biological or genetic predisposition. Research has shown that these constructs are not biologically based and are primarily social constructs (25). This study suggests that racial and ethnic minority populations may have greater risk for SARS CoV-2 infection even after controlling for other social vulnerabilities, testing rates, and the stay-at-home measure. More research is needed to elucidate how minority status and language affects SARS CoV-2 incidence and what other factors are driving these associations.

### Strengths and Limitations

This study has several strengths. The study used secondary data of SARS-CoV-2 incidence, the stay-at-home measure, and social vulnerability. This research contributes to the growing body of evidence on the usefulness of the Social Vulnerability Index and infectious disease outbreaks. Moreover, using anonymized cell phone data to inform public health officials may be a novel approach for emergency response. Few studies have assessed disparities and SARS-CoV-2 in populations with cultural and economic diversity, such as Harris County, and few studies have assessed these relationships at the census tract level.

This study also has several limitations. The SARS-CoV-2 data is from positive cases captured through testing data and does not represent all positive cases in Harris County. The study design is ecological, cannot determine causality or temporality, and does not have a comparison group. Ecological studies such as this study can be prone to ecological fallacy because individuals will be aggregated to the group level and risk factors cannot be linked directly to the outcome. The stay-at-home data may not represent the general population because smartphone use may vary across socio-demographic groups. The stay-at-home data is approximated from smartphone location data and is most likely missing not at random. Though missingness may not be random, the data provider corrects for this (18). Additionally, there may be limitations in the accuracy and precision of the measurements used.

### Public Health Implications

This study found that the CDC's SVI to be significantly associated with SARS-CoV-2 disease incidence even after controlling for testing rate and the stay-at-home measure. By further developing our understanding of the community-level factors that affect infectious disease transmission, public health planners throughout the US can be better prepared for future infectious disease response efforts. This study adds to the growing literature by demonstrating the importance of community-level social vulnerabilities such as minority composition and language barriers associated with poor health outcomes. Understanding why these community-level determinants consistently play such a large role in an individual's health outcomes is key. These findings confirm the usefulness of the SVI in preparing for an infectious disease outbreak. Because the SVI is easily accessible from CDC by census tract, state and local officials can use the index before, during, and after outbreaks occur. The SVI may be used for targeting communities for testing, treatment, health education, and in increasing resources, such as protective equipment, food, and housing assistance. These results also suggest the need to develop culturally and linguistically appropriate mitigation strategies and health education for communities with limited English language proficiency and may face other barriers such as trusting authorities, food insecurity, child care, and housing challenges. Developing partnerships with community-based organizations and community influencers may help with these challenges. Future studies are needed to confirm place-based influences on infectious diseases and how community-based interventions can address disparities in health. Disparities in the current COVID-19 pandemic and any future epidemics or pandemics and their root causes must be addressed through bold policy action and societal investment.

## Data Availability Statement

The original contributions presented in the study are included in the article/supplementary files, further inquiries can be directed to the corresponding author/s.

## Author Contributions

GT conceived the study, analyzed the data, and wrote the manuscript. MO helped conceive the analysis plan and helped write the manuscript. RR produced the map and participated in writing. J-MY helped conceive the analyses, interpret the results, and participated in writing. AR and MB participated in data curation and writing. MP helped to conceive the research plan and participated in writing. EB oversaw the research, participated in writing, and helped in interpretation of the results. All authors contributed to the present manuscript.

## Conflict of Interest

The authors declare that the research was conducted in the absence of any commercial or financial relationships that could be construed as a potential conflict of interest.

## Publisher's Note

All claims expressed in this article are solely those of the authors and do not necessarily represent those of their affiliated organizations, or those of the publisher, the editors and the reviewers. Any product that may be evaluated in this article, or claim that may be made by its manufacturer, is not guaranteed or endorsed by the publisher.

## References

[1] Stokes EK Zambrano LD Anderson KN Marder EP Raz KM El Burai Felix S. Coronavirus Disease 2019 case surveillance — United States, January 22–May 30, 2020. MMWR Morb Mortal Wkly Rep. (2020) 69:759–65. 10.15585/mmwr.mm6924e2 32555134 PMC7302472

[2] Nayak A Islam SJ Mehta A Ko Y-AA Patel SA Goyal A. Impact of social vulnerability on COVID-19 incidence and outcomes in the united states. medrxiv [preprint].medrxiv:2020.04.10.20060962 (2020). 10.1101/2020.04.10.20060962 32511437 PMC7217093

[3] Kim SJ Bostwick W. Social vulnerability and racial inequality in COVID-19 deaths in Chicago. Health Educ Behav. (2020) 47:509–13. 10.1177/1090198120929677 32436405 PMC8183499

[4] Anyane-Yeboa A Sato T Sakuraba A. Racial disparities in COVID-19 deaths reveal harsh truths about structural inequality in America. J Intern Med. (2020) 288:479–80. 10.1111/joim.13117 32452046

[5] Mahajan U V Larkins-Pettigrew M. Racial demographics and COVID-19 confirmed cases and deaths: a correlational analysis of 2886 US counties. J Public Health (Bangkok). (2020) 42:445–7. 10.1093/pubmed/fdaa070 32435809 PMC7313814

[6] Millett GA Jones AT Benkeser D Baral S Mercer L Beyrer C. Assessing Differential impacts of COVID-19 on black communities. Ann Epidemiol. (2020) 47:37–44. 10.1016/j.annepidem.2020.05.003 32419766 PMC7224670

[7] Roberts JD Tehrani SO. Environments, behaviors, and inequalities: reflecting on the impacts of the influenza and coronavirus pandemics in the United States. Int J Environ Res Public Health. (2020) 17:4484. 10.3390/ijerph17124484 32580429 PMC7345270

[8] Zhang CH Schwartz GG. Spatial disparities in coronavirus incidence and mortality in the United States: an ecological analysis as of May 2020. J Rural Heal. (2020) 36:433–45. 10.1111/jrh.12476 32543763 PMC7323165

[9] Pan D Sze S Minhas JS Bangash MN Pareek N Divall P. The impact of ethnicity on clinical outcomes in COVID-19: a systematic review. EClinicalMedicine. (2020) 23:100404. 10.1016/j.eclinm.2020.100404 32632416 PMC7267805

[10] Flanagan BE Gregory EW Hallisey EJ Heitgerd JL Lewis B. A social vulnerability index for disaster management. J Homel Secur Emerg Manag. (2011) 8:3. 10.2202/1547-7355.1792

[11] Banerjee T Nayak AUS. county level analysis to determine If social distancing slowed the spread of COVID-19. Rev Panam Salud Publica. (2020) 44:e90. 10.26633/RPSP.2020.90 32636878 PMC7334824

[12] Rubin D Huang J Fisher BT Gasparrini A Tam V Song L. The Association of social distancing, population density, and temperature with the SARS-CoV-2 instantaneous reproduction number in counties across the United States. medRxiv [preprint].medrxiv:2020.05.08.20094474 (2020). 10.1101/2020.05.08.20094474 32701162 PMC7378754

[13] Biggs EN Maloney PM Rung AL Peters ES Robinson WT. The relationship between social vulnerability and COVID-19 incidence among louisiana census tracts. Front Public Heal [Internet]. (2021) 8:617976. 10.3389/fpubh.2020.617976 33553098 PMC7856141

[14] Karmakar M Lantz PM Tipirneni R. Association of social and demographic factors with COVID-19 incidence and death rates in the US. JAMA Netw Open. (2021) 4:e2036462. 10.1001/jamanetworkopen.2020.36462 33512520 PMC7846939

[15] Oluyomi AO Gunter SM Leining LM Murray KO Amos C. COVID-19 community incidence and associated neighborhood-level characteristics in Houston, Texas, USA. Int J Environ Res Public Health. (2021) 18:1–16. 10.3390/ijerph18041495 33557439 PMC7915818

[16] Khazanchi R Beiter ER Gondi S Beckman AL Bilinski A Ganguli I. County-level association of social vulnerability with COVID-19 cases and deaths in the USA. J General Internal Med. (2020) 35:2784–7. 10.1007/s11606-020-05882-3 32578018 PMC7311112

[17] QuickFacts Harris County Texas. United States Census Bureau (2019).

[18] Levin R Chao DL Wenger EA Proctor JL. Insights into population behavior during the COVID-19 pandemic from cell phone mobility data and manifold learning. Nat Comput Sci [Internet]. (2021) 1:588–97. 10.1038/s43588-021-00125-9 PMC10766515 38217135

[19] U.S. Consumer Activity During COVID-19 Pandemic [Internet]. (2020). Available online at: https://www.safegraph.com/dashboard/covid19-commerce-patterns (accessed August 22, 2021).

[20] Jay J Bor J Nsoesie EO Lipson SK Jones DK Galea S. Neighbourhood income and physical distancing during the COVID-19 pandemic in the United States. Nat Hum Behav. (2020) 4:1294–302. 10.1038/s41562-020-00998-2 33144713 PMC8107986

[21] Piza EL. Using Poisson and Negative Binomial Regression Models to Measure the Influence of Risk on Crime Incident Counts. New Jersey, NJ: Rutgers Center (2012).

[22] Sydenstricker E. The incidence of influenza among persons of different economic status during the epidemic of 1918. 1931. Public Health Rep. (2006) 121(Suppl. 1):154–70. 10.2307/4579923 16550779

[23] Shippee TP Akosionu O Ng W Woodhouse M Duan Y Thao MS. COVID-19 pandemic: exacerbating racial/ethnic disparities in long-term services and supports. J Aging Soc Policy [Internet]. (2020) 32:1–11. 10.1080/08959420.2020.1772004 32476614 PMC9152947

[24] Hawkins D. Social determinants of covid-19 in massachusetts, United States: an ecological study. J Prev Med Public Heal. (2020) 53:220–7. 10.3961/jpmph.20.256 32752590 PMC7411251

[25] Goodman AH. Why genes don't count (for racial differences in health). Am J Public Health. (2000) 90:1699–702. 10.2105/AJPH.90.11.1699 11076233 PMC1446406

